# Insights into diastolic function analyses using cardiac magnetic resonance imaging: impact of trabeculae and papillary muscles

**DOI:** 10.1186/s13244-021-01104-4

**Published:** 2021-11-03

**Authors:** Bjoern P. Schoennagel, Kai Müllerleile, Enver Tahir, Jitka Starekova, Regine Grosse, Jin Yamamura, Peter Bannas, Gerhard Adam, Roland Fischer

**Affiliations:** 1 Department of Diagnostic and Interventional Radiology and Nuclear Medicine, University Medical Center Hamburg-Eppendorf, Martinistrasse 52, 22609 Hamburg, Germany; 2 Department of Cardiology, University Heart and Vascular Center, University Hospital Hamburg-Eppendorf, Martinistrasse 52, 22609 Hamburg, Germany; 3 Department of Pediatric Hematology and Oncology, University Medical Center Hamburg-Eppendorf, Martinistrasse 52, 22609 Hamburg, Germany; 4 UCSF Benioff Children’s Hospital, Oakland, USA

**Keywords:** Diastolic function, Trabeculae and papillary muscles, Cardiovascular magnetic resonance

## Abstract

**Background:**

This cardiovascular magnetic resonance (CMR) study investigates the impact of trabeculae and papillary muscles (TPM) on diastolic function parameters by differentiation of the time-volume curve. Differentiation causes additional problems, which is overcome by standardization.

**Methods:**

Cine steady-state free-precession imaging at 1.5 T was performed in 40 healthy volunteers stratified for age (age range 7–78y). LV time-volume curves were assessed by software-assisted delineation of endocardial contours from short axis slices applying two different methods: (1) inclusion of TPM into the myocardium and (2) inclusion of TPM into the LV cavity blood volume. Diastolic function was assessed from the differentiated time-volume curves defining the early and atrial peaks, their filling rates, filling volumes, and further dedicated diastolic measures, respectively.

**Results:**

Only inclusion of TPM into the myocardium allowed precise assessment of early and atrial peak filling rates (EPFR, APFR) with clear distinction of EPFR and APFR expressed by the minimum between the early and atrial peak (EA_min_) (100% vs. 36% for EA_min_ < 0.8). Prediction of peak filling rate ratios (PFRR) and filling volume ratios (FVR) by age was superior with inclusion of TPM into the myocardium compared to inclusion into the blood pool (*r*^2^ = 0.85 vs. *r*^2^ = 0.56 and *r*^2^ = 0.89 vs. *r*^2^ = 0.66). Standardization problems were overcome by the introduction of a third phase (mid-diastole, apart from diastole and systole) and fitting of the early and atrial peaks in the differentiated time-volume curve.

**Conclusions:**

Only LV volumetry with inclusion of TPM into the myocardium allows precise determination of diastolic measures and prevents methodological artifacts.

## Key points

- Trabeculae and papillary muscles (TPM) have impact on diastolic function assessed by MRI.
- Inclusion of TPM into the myocardium allows precise determination of filling rates.
- Inclusion of TPM into the myocardium offer a higher degree of standardization.
- A third cardiac phase (mid-diastole) is important for precise analysis.

## Background

The relevance of CMR-derived diastolic measures in day-to-day clinical practice is increasingly recognized [1–3]. To ensure consistent quality and reliability of CMR studies, agreement on specific standards for post-processing is needed [4]. However, the influence of trabeculae and papillary muscles (TPM), e.g., considering TPM either as part of the LV myocardium or the LV blood pool, on diastolic function analyses has not been addressed to our knowledge.

Diastolic function can be assessed by cine CMR applying the differentiated time-volume curve [1, 5–7]. Assessment of the time-volume curve requires LV volumetry that can be performed by two different approaches, either inclusion of TPM into the myocardium or into the LV blood pool. There is a well-known impact of this decision on global systolic LV parameters, which may influence clinical management [8–10]. As TPM are myocardial tissue, they should ideally be considered as part of the LV myocardium [4]. Due to practical considerations, TPM are often included into the blood pool volume, which is an accepted approach [4]. Instead, there is no practical guideline concerning the methodological approach for TPM in the assessment of diastolic function. This resulted in inconsistent LV volumetries with either inclusion of TPM into the blood pool [7, 11, 12] or myocardium [13, 14], or handling of TPM was not sufficiently described [6, 15, 16]. In addition, to our knowledge there is no systematic evaluation of methodological factors influencing time-volume curve derived diastolic function analysis.

The aim of this study was to investigate the impact of TPM on diastolic function analyses from CMR-derived time-volume curve in healthy controls and to give insight into the methodology of time-volume curve analysis for assessment of diastolic function.

## Materials and methods

### Patients

CMR was performed in 40 healthy volunteers (12 female/28 male, mean age 37 ± 19 y, range 7–78 y), some of them were part of a former study. No participant had history of cardiac disease and no systolic trans-mitral blood flow as sign of mitral valve regurgitation was observed in 4-chamber view cine images. Four individuals underwent 2–4 repeated CMR scans (between 40 and 156 months).

The institutional committee on human research approved the study, and all volunteers gave their written informed consent prior to examination.

### CMR protocol

CMR was performed on a 1.5 T MR system (Symphony, Siemens Healthcare, Erlangen, Germany) using a four-element phased array coil. Single slice acquisitions were performed with breath hold and retrospectively ECG-gated cine SSFP sequences. Phase encoding steps (segments per view) were reduced for higher heart rates. Specifically, cardiac function was assessed from short axis cine-series (TE = 1.6 ms, TR = 50 ms, FA = 65°, bandwidth = 965 Hz/pixel, in-plane resolution = 1.5 × 1.5 mm^2^, slice thickness = 6, gap = 0/6 mm, cardiac phases = 25).

### LV volumetry and diastolic function

LV volumetry was assessed by two radiologists with more than 10 years of CMR experience. LV volumetry was performed with: (1) inclusion of TPM into the myocardium and (2) inclusion of TPM into the LV blood pool*.*

**Fig. 1:**
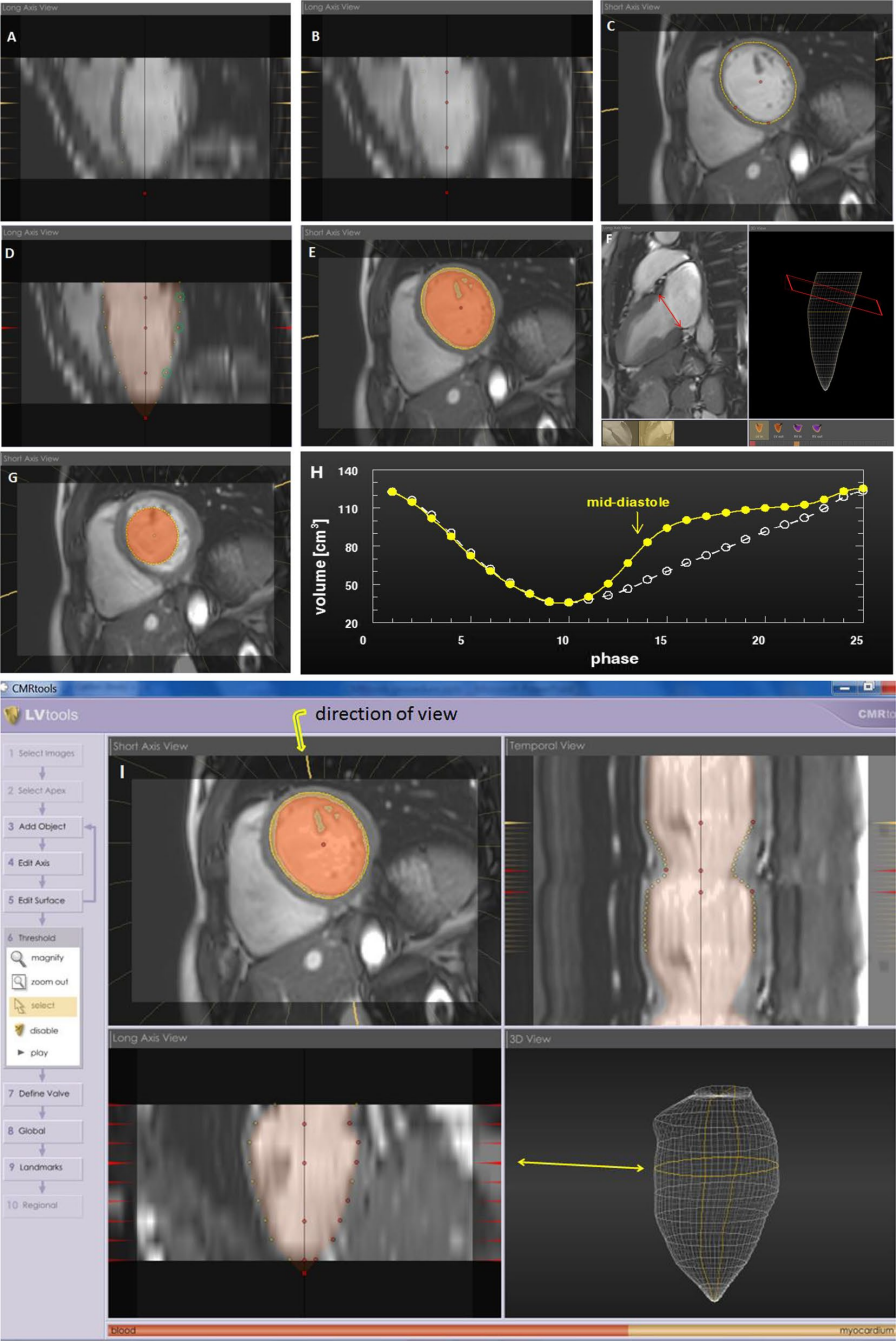
LV volumetry using CMRtools. The longitudinal LV main axis (black line in **A**) is defined from three representative short axis slices. Red points along the longitudinal main axis (**B**) illustrate localization of short axis slices where the longitudinal axis was set (diastole and systole, respectively). The LV endocardial contour (yellow circumscript in **C**) is set by manual definition of endocardial landmarks (red points in **C**) and propagating (yellow points along the endocardium in **C**) in a diastolic (shown) and systolic mid-papillary short axis slice. This contour is propagated for the other cardiac phases and the short axis stack. The resulting LV blood volume (including TPM) is visualized in long axis view (red color in **D**), and endocardial contour can be controlled (green circles in **D**). Exclusion of TPM (yellow in **E**) from the LV blood pool (red in **E**) is automatically provided by the software. Definition of mitral valve plane is done in 2- and 4-chamber views in diastole and systole (red line with arrows in **F**, left) to separate blood volumes of the atrium and ventricle. Mitral valve plane is shown (red rectangle in **F**, right) in a 3D geometric model of the LV. A third cardiac phase (mid-diastole) is selected in a mid-papillary short axis slice to manually correct for potential mismatch of the true and the propagated endocardial border (yellow circumscript of red area in **G**). The impact of considering this third cardiac phase becomes obvious comparing the time-volume curves without (white curve in **H**) and with (yellow curve in **H**) adjustment of endocardial borders in the third phase. In a final fine-tuning step endocardial borders (yellow points in **I**) were controlled and adjusted where necessary in all three cardiac phases (diastole, systole, mid-diastole). Yellow arrow (upper left) indicates view direction of the temporal view (quasi M-Mode, upper right)

LV end-diastolic volumes (EDV), end-systolic volumes (ESV), EF and myocardial mass (M) were determined by manual delineation of endocardial and epicardial borders in end-systolic, end-diastolic and mid-diastolic short axis views using dedicated software (CMRtools®, v. 2010, Cardiovascular Imaging Solutions Ltd, Cambs, UK) (Fig. 1). LV blood and myocardial volumes were calculated for each of the acquired 25 phases of the cardiac cycle by the following steps out of CMRtools’ workflow, each step characterized by a set of four images (Fig. 1I):

- The longitudinal main axis of the LV chamber was set by defining the central symmetry axis of the LV chamber in three representative short axis slices (i.e. basal, mid-papillary and apical) in diastole and systole, respectively (Fig. 1A, B).
- Delineation of LV endocardial borders was performed in a mid-papillary short axis slice in diastole and systole, respectively (Fig. 1C). The software automatically propagates endocardial contours for the other slices and phases of the short axis stack, respectively.
- The resulting LV blood volume (including TPM) can be visualized in red color by activating the “shading” function, and its correct contour can be controlled as indicated by green circles in the long axis view of Fig. 1D.
- Exclusion of TPM from the blood pool is automatically provided by the software based on different signal intensities of myocardium/TPM and blood (Fig. 1E). The individual threshold for precise identification of TPM can manually be adjusted on a color contrast scale (see ruler bar at the bottom of Fig. 1I) and was set in a mid-papillary diastolic short axis slice. The scale has to be moved until TPM are excluded from the LV blood pool. This step of TPM exclusion with threshold adjustment takes only several seconds.
- The mitral valve plane was manually defined in diastolic and systolic 4- and 2-chamber views (see red arrow in Fig. 1F, left). This results in an oblique plane in the three-dimensional LV model (red rectangle in Fig. 1F, right) for precise separation of blood volumes of the left ventricle and atrium.
- A third cardiac phase (mid-diastolic) was selected in a mid-papillary short axis slice, where the propagated endocardial contour showed the largest deviation from the real LV contour (Fig. 1G). Endocardial borders were manually corrected for this cardiac phase, resulting in a significantly different time volume curve (Fig. 1H, yellow symbols) in comparison with the curve without consideration of a third phase (Fig. 1H, white circles).
- Finally, a fine-tuning procedure of the LV endocardial contour was performed in all three (diastole, systole, mid-diastole) phases by manually adjusting the endocardial border points (Fig. 1I, upper left) where necessary. This procedure results in a three-dimensional wire model showing all short axis slices below the basal plane (Fig. 1I, bottom right).

**Fig. 2:**
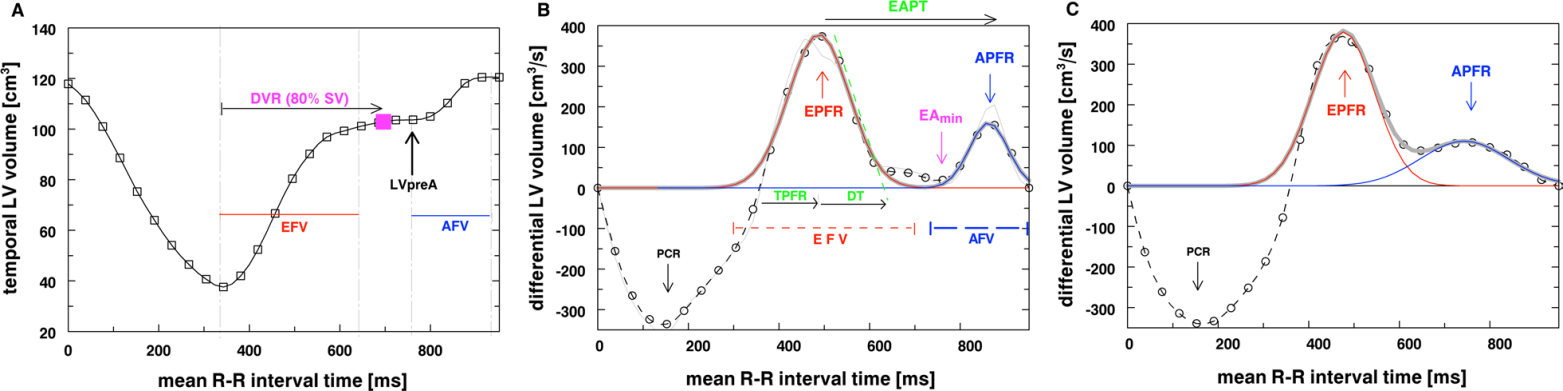
Temporal LV volume and derived diastolic parameters in a healthy volunteer. **A** Temporal LV volume (squares) by inclusion of TPM into the LV myocardium and derived diastolic parameters (LV_preA_, DVR). **B** Temporally differentiated LV volume (thin line) and after smoothing (circles, dashed line) with derived diastolic early and atrial peak filling rate (EPFR and APFR) indicated by arrows. Early (EFV) and atrial (AFV) peak filling volumes are determined as areas from a Gaussian dual-peak fit (grey, red, and blue line). **C** Disabling the tissue-blood threshold (= inclusion of TPM into the blood pool) leads to overlapping early and atrial peaks

From the time-volume curve observables (R-R time intervals (phases), temporal TPM masses and blood volumes), the LV diastolic function parameters PFRR, DVR, etc., can be semi-automatically assessed in an EXCEL template. The only manual adjustment has to be made for the minimum between the early and atrial peak (EA_min_) (Fig. 2B).

The early and atrial (late) filling volumes (EFV and AFV) refer to the early diastolic LV filling due to passive LV relaxation, and the late LV filling due to active contraction of the left atrium (Fig. 2A). However, there is more information in this time pattern of ventricular volumes, which is revealed after differentiation. The temporally differentiated LV time-volume curve usually results in three peaks characterized by the systolic peak contraction rate (PCR) as well as the diastolic early (EPFR) and atrial (APFR) peak filling rate (Fig. 2B, C). In order to avoid artificial differentiation peaks, the data were smoothened by their next neighbors. As result, these two separated diastolic peaks can be fitted by a Gaussian dual-peak fit resulting in the early and atrial filling volumes (EFV, AFV) (Fig. 2B). The calculated filling volume ratio (FVR = EFV / AFV) is the corresponding volumetric (= area) counterpart to the phase-specific (= peak) peak filling rate ratio (PFRR = EPFR/APFR).

The EPFR and APFR assessed by CMR reflect the early (E) and atrial (A) transmitral peak filling velocities determined by echocardiography [13]. In analogy to echocardiography (E/A ratio), the peak filling rate ratio (PFRR = EPFR/APFR) is the equivalent to characterize diastolic filling patterns using CMR. Here, we have to point out that assessment of E/A ratios (PFRR, FVR) by CMR relies on filling volumes, while echocardiographic assessments address velocity measurements across the mitral valve [15].

The time interval between the early atrial phase peak and the atrial phase peak normalized to the RR-cycle (heart rate) is characterized by EAPT%, while EA_min_ describes the distinctiveness of the atrial peak relative to the minimum rate between EPFR and APFR. The corresponding temporal volume is LV_preA_.

Further echocardiographic-derived diastolic indices that were determined included the time to peak filling rate (TPFR) and deceleration time (DT), describing the rise and decline of the early diastolic peak. The diastolic volume recovery (DVR) is defined according to Kawaji et al. [13], i.e., as proportion of diastole required to recover 80% of systolic volume, either absolute (DVR in ms) or as percentage of diastole (DVR%) (Fig. 2A).

Except for the deceleration time (DT%), all diastolic parameters were assessed from both volumetric approaches with either inclusion of TPM into the LV myocardium or the LV blood pool.

The influence of temporal resolution on diastolic measures was studied at temporal resolutions of 12 ms (= 64 phases), 16 ms (= 50 phases) and 30 ms (= 25 phases) in one healthy control (26 y).

### Statistical and data analysis

Since many of the respective parameters had a skewed distribution (skewness > 1.0 ± 0.2), we applied nonparametric statistics reaching statistical significance by two-sided *p* < 0.05: median, 95% range, paired Wilcoxon test and Spearman rank correlation (*r*_S_) test (STATISTICA, Stat-Soft Inc., Tulsa, USA).

For adjustment (prediction) of diastolic LV parameters as a function of age, we fitted exponential models to the data using the Marquardt–Levenberg fit algorithm of Slide Write Plus for Windows (Advanced Graphics Software Inc., Encinitas, CA, USA), which resulted in adjusted coefficients of determination (*r*^2^), curve parameters ± standard errors. Similarly, the diastolic filling rates were fitted by a Gaussian twin peak model of type a0**·**exp(− 0.5**·**((*x* − a2)/a1)^2^) with a0 = amplitude (cm^3^/s), a1 = peak width/2 (ms), and a2 = peak position (ms) (fitted by Slide Write). The early or atrial filling volumes can then be calculated from the fitted parameters by a0**·**a1/1000**·**√(2*π*).

A semi-automatic EXCEL analysis template was developed for calculating critical time points within the differentiated time-volume curve (systole, early and atrial peaks) and derived non-trivial diastolic parameters (LV_preA_, DVR, EAPT, EA_min_, TPFR, DT, PFRR, FVR). This template can be obtained from the authors.

## Results

Inclusion of TPM into the LV myocardium resulted in two sharply separated diastolic peaks of the differential time-volume curve (Fig. 2B). In contrast, inclusion of TPM into the blood pool revealed overlapping diastolic early and atrial peaks in the majority of patients (Fig. 2C). The diastolic early and atrial peak filling rates EPFR and APFR and the corresponding early and atrial filling volumes EFV and AFV, respectively, were only detectable by inclusion of TPM into the myocardium (Fig. 2B). In agreement, inclusion of TPM into the myocardium yielded a distinction of EPFR and APFR in 98% of volunteers as expressed by EA_min_ < 0.8. Instead, with inclusion of TPM into the blood pool this criterion was achieved in only 38% of volunteers. Similarly, inclusion of TPM into the LV myocardium demonstrated lower time-volumes, LV_preAi_, at EA_min_ compared with inclusion of TPM into the blood pool. In addition, inclusion of TPM into the myocardium generated smaller early (EPW) and atrial (APW) peak widths of the Gaussian fitted peaks. The wider separation of peaks due to inclusion of TPM into the myocardium leads also to a larger IQR (spreading) of DVR (DVR%), while no difference was found for TPFR. The deceleration time (DT) could reliably be determined only with inclusion of TPM into the myocardium. The temporal separation of the early and atrial peak is described by the time interval EAPT that was significantly higher with inclusion of TPM into the myocardium (36% vs. 24%, *p* < 10^–3^). Multiple regression resulted in an inverse relationship (*r*^2^ = 0.43) of EAPT with heart rate (*p* < 10^–4^) and age (*p* = 0.002), however, including TPM into the blood pool only left HR as significant variable (*p* = 0.02). A summary and comparison of all diastolic parameters assessed by both inclusion of TPM into the myocardium and blood pool are provided in Table 1.

**Table 1:** Diastolic function parameters by inclusion of TPM into the myocardium and into the blood pool

Parameter	TPM-myo				TPM-blood				Paired Wilcoxon test
	Median	IQR	2.5%	97.5%	Median	IQR	2.5%	97.5%	*p*
*n* (m/f)	40 (28/12)								
Age (y)	31	23	9	76					
BSA (m^2^)	1.79	0.31	0.98	2.08					
HR (bpm)	69	9	56	91					
LVEF (%)	68	5	57	79	62	7	54	71	*p* < 10^–4^
LVpreAi (mL/m^2^)	62	22	41	89	75	24	48	103	*p* < 10^–4^
EAmin	0.20	0.29	−0.03	0.80	0.97	0.60	0.17	1.27	*p* < 10^–4^
EPFRi (mL/s/m^2^)	227	114	124	411	214	87	112	351	*p* < 10^–4^
EPFR/SV (1/s)	4.8	1.5	2.9	7.6	3.9	0.9	2.7	5.4	*p* < 10^–4^
APFRi (mL/s/m^2^)	118	73	53	185	92	37	53	185	0.025
PFRR = EPFR/APFR	2.4	1.6	0.7	7.1	2.2	1.2	0.9	4.5	0.3
EFVi (mL/s/m^2^)	37	11	23	50	41	12	21	63	*p* < 10^–4^
AFVi (mL/s/m^2^)	12	7	5	19	14	7	5	22	*p* < 10^–3^
FVR = EFV/AFV	3.4	1.8	1.1	9.6	3.1	1.9	1.1	7.8	0.08
DVR (ms)	339	153	178	534	291	73	204	456	0.044
DVR (%)	69	22	33	83	56	13	43	81	0.023
EPW (ms)	126	26	84	167	161	35	98	199	*p* < 10^–4^
APW (ms)	84	19	57	110	128	54	68	225	*p* < 10^–4^
EAPT (%)	36	9	22	48	24	20	16	48	*p* < 10^–3^
TPFR (ms)	146	35	112	188	140	29	108	193	0.4
DT (ms)	124	19	104	192					
DTj (%)	15.2	3.4	11	27					

TPM-myo, inclusion of trabeculae and papillary muscles into the myocardium; TPM-blood, inclusion of trabeculae and papillary muscles into the blood pool; BSA, body surface area; HR, heart rate; LVEF, LV ejection fraction; LVpreA, time-volume at EA_min_; EA_min_, minimum between early and atrial peak relative to APFR; DVR, diastolic volume recovery; EPFR, early peak filling rate; APFR, atrial peak filling rate; PFRR, peak filling rate ratio; EFV, early filling volume; AFV, atrial filling volume; FVR, filling volume ratio; EPW, early peak width; APW, atrial peak width; EAPT%, early to atrial peak time relative to HR; TPFR, time to peak filling rate; DT, deceleration time; i, indexed parameter

The relative cardiac tissue-blood threshold, which is proportional to the amount of TPM, ranged from 10 to 41% of the LV blood pool.

With inclusion of TPM into the myocardium, highly significant correlations with age were found for most assessed diastolic parameters, especially for PFRR, FVR, DVR%, DT, and LV_preAi_ (all *p* < 10^–3^). This was in contrast to TPM included into the blood pool. No correlation with any of these diastolic parameters was found for the systolic EF.

**Fig. 3:**
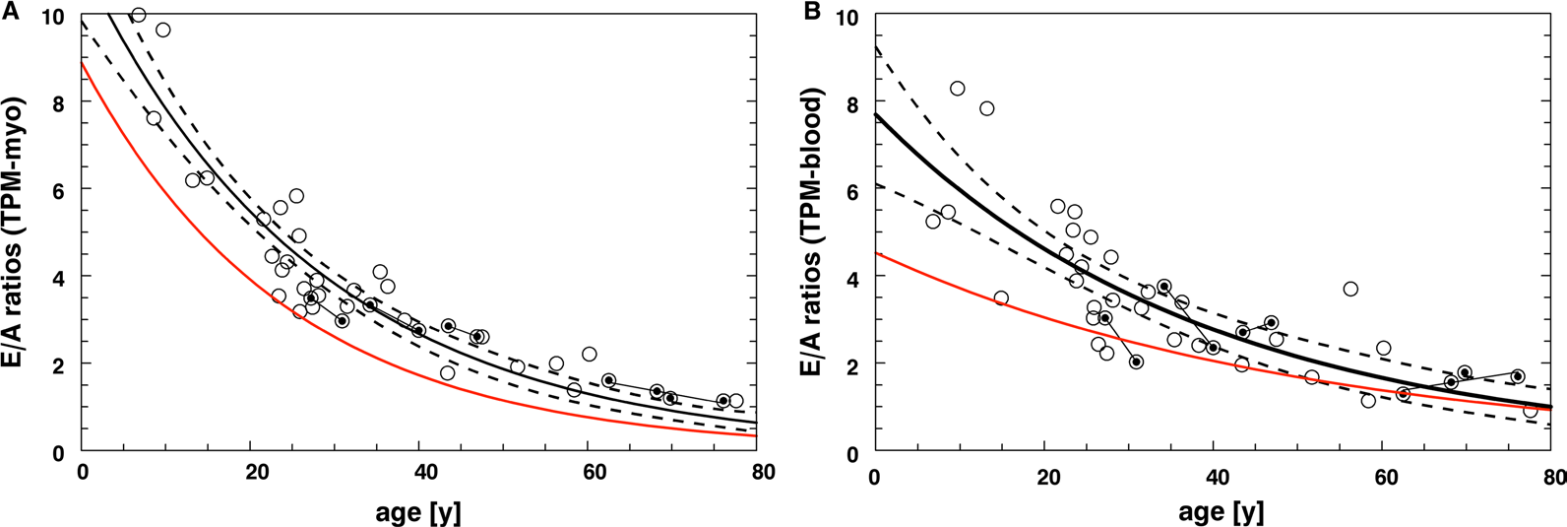
Exponential relationship of early to atrial ratios (E/A) as function of age in healthy volunteers. Gaussian fitted filling volumes (FVR, solid line, 95% confidence interval by dashed lines), and peak filling rates (PFRR, red line) were determined by (**A**) inclusion of trabeculae and papillary muscles into the myocardium (TPM-myo) and (**B**) into the blood pool (TPM-blood). With TPM-myo, coefficients of determination were significantly higher for FVR (*r*^2^ = 0.89 vs. *r*^2^ = 0.66) and PFRR (*r*^2^ = 0.85 vs. *r*^2^ = 0.56). Intra-individual follow-up measurements (*n* = 2–4) in four subjects are connected by trend lines

An exponential model was applied for prediction of PFRR and FVR as a function of age. Prediction by age was superior with inclusion of TPM into the myocardium compared to inclusion into the blood pool, expressed by higher coefficients of determination for PFRR (*r*^2^ = 0.85 vs. *r*^2^ = 0.56) and FVR (*r*^2^ = 0.89 vs. *r*^2^ = 0.66) (Fig. 3A, B). Using Gaussian fitted filling volumes, we obtained the relationship FVR = a0·exp(− age/a1) with a0 = 11.2 ± 0.6, a1 = 27.9 ± 1.8 y, and a standard error (SE) of 0.72 (Fig. 3A, Table 2). For TPM included into the myocardium, all significant function parameters stratified for age are shown in Table 2.

**Table 2:** Diastolic parameters with TPM included into myocardium for calculating normal values as significant function of age

Parameter	*p*	Function of age (= *x*)	a0	a1	a2	*r*^2^	SEE
LVpreAi	7.6E-04	a0 + a1∙x	77.2	− 0.42		0.28	12.1
DVR%	*p* < 10^–6^	a0 (1 − exp(− x/a1)) + a2	69.1	30.8	18.6	0.64	9.50
EPFR/SV	*p* < 10^–6^	a0∙exp(− x/a1)	7.45	82.3		0.64	0.76
APFRi	*p* < 10^–6^	a0∙exp(− x/a1)	7.45	82.3		0.64	0.76
PFRR	*p* < 10^–6^	a0∙exp(− x/a1)	8.9	24.4		0.85	0.66
FVR	*p* < 10^–6^	a0∙exp(− x/a1)	11.2	27.9		0.89	0.72
TPFR (ms)	4.5E−04	a0 (1 − exp(− x/a1)) + a2	74.7	21.5	90.2	0.30	19.1
DT (ms)	4.0E−06	a0 (1 − exp(− x/a1)) + a2	63.9	22.4	84.6	0.14	24.2

LVpreA, time-volume at EA_min_; EA_min_, minimum between Early and Atrial peak relative to APFR; DVR%, diastolic volume recovery (percentage to diastole); EPFR/SV, early peak filling rate/stroke volume; APFR, atrial peak filling rate; PFRR, peak filling rate ratio; FVR, filling volume ratio; TPFR, time to peak filling rate; DT, deceleration time; i, indexed parameter

Spearman rank correlation probabilities (*p*), coefficients a0–a2, coefficient of determination r^2^, and standard error of estimate (SEE)

Inter-operator variability between two analysts was tested by Bland–Altman plots in 15 controls. Agreement was exemplarily found for tissue-blood threshold, LVEF, PFRR, and FVR with insignificant bias and within ± 95% absolute limits of agreement (− 95% LoA, + 95% LoA) as (− 9.1%, 6.1%), (− 6.2%, 6.5%), (− 0.8, 1.1) and (− 1.9, 1.5), respectively.

Decreasing temporal resolutions from 12 to 30 ms assessed in one healthy volunteer resulted in declined EPFR and APFR, while PFRR increased, respectively (from 2.19 to 3.03). The isovolumetric relaxation time (IVRT, defined as the time between onset and end of isovolumetric relaxation [15]) could clearly be observed at 12 ms. In the heart rate (HR) interval of our population from 54 bpm (resolution 44 ms) to 91 bpm (25 ms), the following parameters revealed significant correlation with heart rates: EA_min_ (*p* < 0.0002), DVR (*p* < 0.0004), EPFR (*p* < 0.02), EAPT% (*p* < 0.00005).

## Discussion

This CMR study investigated the impact of TPM on diastolic function analyses derived from the LV time-volume curve. Only inclusion of TPM into the myocardium provided clear demarcation of diastolic shape characteristics and thus precise determination of diastolic measures as the peak filling rates. Standardization problems were overcome by the introduction of a third cardiac phase (mid-diastole) and fitting of the early and atrial peaks in the differentiated time-volume curve. Knowledge of factors influencing CMR-derived diastolic function analysis is necessary to avoid methodological susceptibilities.

The differentiated LV time-volume curves from our healthy volunteers indicate that only inclusion of TPM into the LV myocardium allows reliable identification of diastolic peak filling rates EPFR and APFR as well as other dedicated diastolic parameters. In contrast, with inclusion of TPM into the LV blood pool definition of APFR and EA_min_ was not sufficient and DT was not assessable. Estimation of peak filling rates was also impaired with inclusion of TPM into the blood pool, resulting in only moderate correlation of the PFRR assessed by either inclusion of TPM into the myocardium or blood pool. Fundamental discrepancies between both volumetric approaches were reflected by diastolic parameters that are sensitive to special shape characteristics of the differentiated time-volume curve: sensitivity for EA_min_ < 0.8 was 98% with inclusion of TPM into the myocardium compared to 38% with inclusion into the blood pool. Similar results were reported by Kawaji et al. with 76% sensitivity for distinction of EPFR and APFR including TPM into the myocardium [13]. Also, the EAPT was significantly longer with inclusion of TPM into the myocardium, reflecting distinct separation of the early and atrial peaks compared to overlapping peaks by including TPM into the blood pool. The results of this study illustrate that TPM have a significant impact on quantitative diastolic measures. We suggest LV volumetry with inclusion of TPM into the LV myocardium as the preferred method in the assessment of diastolic function by CMR-derived time-volume curves to ensure consistent quality.

In our healthy volunteers, the PFRR and FVR could be predicted by an exponential function of age. Therefore, adjustment of these indices to age (e.g., by Z-scores) is crucial in the clinical evaluation of diastolic function. Age dependency of PFRR and FVR revealed another important methodological aspect of post-processing. Theoretically, peak filling rates (= max. peak heights) are more susceptible to methodological artifacts than filling volumes, as they refer to a single cardiac phase (= peak) instead of early and atrial filling intervals (= areas). With inclusion of TPM into the myocardium, we observed similar and high prediction for PFRR (peaks) and FVR (volumes) by age. Instead, with inclusion of TPM into the blood pool PFRR was more susceptible compared to FVR and prediction was lower. Therefore, inclusion of TPM into the myocardium demonstrated to be the more robust approach, not being influenced by methodological artifacts. Using this approach for LV volumetry, estimation of PFRR provides a practical approach as it is easy to determine and revealing similar accuracy compared to filling volumes.

Besides TPM, there are different aspects to consider in the understanding of diastolic function analyses by CMR-derived time-volume curves. Krishnamurthy et al. demonstrated significant reduction of peak filling rates at lower temporal resolutions [15]. We observed the same behavior with altered PFRR of about 25% applying variable temporal resolutions from 12 to 30 ms in a single healthy subject. In addition, isovolumetric relaxation time (IVRT) could only be resolved at times <  = 12 ms, which agrees with former observations [15]. However, the standard diastolic parameters EPFR, APFR and PFRR and the corresponding filling volumes EFV, AFV and FVR were not substantially affected by HR (= temporal resolution) < 80 bpm. The influence of the HR seems relevant, especially for heart rates > 80 bpm (e.g., typically in children) where the resolution to depict the early and atrial peak relative to EAPT is getting worse.

Diastolic parameters of our healthy volunteers were in agreement with healthy controls of other CMR studies performing inclusion of TPM into the LV myocardium. Mean PFRR of controls in the third decade was 3.0 (range 1.7–4.4) and similar to our age-matched volunteers with 3.3 (range 1.8–6.3) [14]. Also, the PFRR in older healthy controls of a different study was in agreement with our age-matched healthy volunteers (2.3 ± 1.0 vs. 2.2 ± 1.0) [16]. PFRR, TPFR and DVR were comparable with 3.1 ± 2.4, 174 ± 119 ms and 65 ± 16% in an older age-matched control group (age 50 ± 10 y) [6]. Also, 20 older woman (age 54 ± 9 y) of a different study group revealed comparable results for EPFR indexed to stroke volumes (4.2 ± 0.6 vs. 4.6 ± 0.9) and TPFR (200 ± 20 ms vs. 152 ± 21 ms), respectively [12]. The similar results of these inter-study comparisons support the idea that diastolic function analyses performed by inclusion of TPM into the myocardium allows reproducible estimation of diastolic measures.

CMR is considered as a gold standard clinical technique to assess cardiac function and volumes. Our analysis of healthy volunteers provides diastolic parameters as function of age to calculate reference values for healthy controls performing LV volumetry with inclusion of TPM into the myocardium. Our data provide useful clinical and research utility as the diastolic filling rates EPFR and APFR and their ratio PFRR are the most commonly assessed parameters in the evaluation of CMR-derived diastolic function. There are few reports providing age-dependent reference values of CMR-derived diastolic function in healthy volunteers [14, 17]. LV filling profiles assessed from the first derivate of the time-volume curve are increasingly used for the determination of diastolic parameters [6, 18, 19]. The necessity for standardized post-processing procedures is an ongoing issue and a prerequisite to ensure consistent quality and reproducibility of CMR reports [4]. Furthermore, due to ongoing publications and introduction of new techniques, reference values require frequent update and integration [20]. We think that knowledge of the influence of TPM on diastolic function analyses can enhance utility and precision of CMR studies and might also help in the evaluation of diastolic dysfunction as, e.g., regularly seen in patients with transfusion dependent anemias with or without iron overload [21].

A potential limitation of this study is the relatively small group of healthy volunteers. However, we stratified patients as a function of age instead of providing reference ranges per decade. Although FVR showed similar exponential relationships with age for females and males (*r*^2^ = 0.94 and 0.89), no significant gender differences (*p* = 0.7 by discriminant analysis) could be observed between the two groups in contrast to former work [14, 17]. However, this was an approach primarily analyzing methodological effects on diastolic parameters. A further limitation arises from the time-consuming approach of LV volumetry and analyses steps of approximately 20 min. Although this is more a practical concern of the method (and not the study itself) itself, it may limit transfer and application of this method into the clinical workflow. In addition, there is no comparison and validation of diastolic function derived from LV volumetry with other established methods, e.g., phase-contrast MRI (transmitral flow) or echocardiography.

## Conclusions

This study in healthy volunteers investigated the impact of TPM on CMR diastolic function analyses derived from the LV time-volume curve and gives insight into methodological considerations. Standardized post-processing with inclusion of TPM into the myocardium is necessary for precise determination of diastolic measures. Our data contribute to the understanding and standardization of post-processing and provide reference values of time-volume curve derived diastolic parameters as a function of age in healthy subjects.

## Data Availability

The datasets generated and analyzed during the current study are not publicly available due to local restrictions of data protection but are available from the corresponding author on reasonable request.

## Abbreviations

AFV: atrial filling volume
APFR: atrial peak filling rate
APW: atrial peak width
BSA: body surface area
DT: deceleration time
DVR (%): diastolic volume recovery (percentage to diastole)
EA_min_: minimum between early and atrial peak relative to APFR
EAPT (%): early to atrial peak time (relative to HR)
EFV: early filling volume
EPFR: early peak filling rate
EPW: early peak width
FVR: filling volume ratio
HR: heart rate
LVEF: left ventricular ejection fraction
LVpreA: time-volume at EA_min_
PCR: peak contraction rate
PFRR: peak filling rate ratio
TPFR: time to peak filling rate
TPM: trabeculae and papillary muscles

## References

[1] Caudron J, Fares J, Bauer F, Dacher J-N (2011). Evaluation of left ventricular diastolic function with cardiac MR imaging. Radiographics.

[2] Chamsi-Pasha MA, Zhan Y, Debs D, Shah DJ (2020). CMR in the evaluation of diastolic dysfunction and phenotyping of HFpEF. JACC Cardiovasc Imaging.

[3] Leong DP, De Pasquale CG, Selvanayagam JB (2010). Heart failure with normal ejection fraction: the complementary roles of echocardiography and CMR imaging. JACC Cardiovasc Imaging.

[4] Schulz-Menger J, Bluemke DA, Bremerich J (2020). Standardized image interpretation and post-processing in cardiovascular magnetic resonance: 2020 update. J Cardiovasc Magn Reson.

[5] Westwood MA, Wonke B, Maceira AM (2005). Left ventricular diastolic function compared with T2* cardiovascular magnetic resonance for early detection of myocardial iron overload in thalassemia major. J Magn Reson Imaging.

[6] Mendoza DD, Codella NCF, Wang Y (2010). Impact of diastolic dysfunction severity on global left ventricular volumetric filling: assessment by automated segmentation of routine cine cardiovascular magnetic resonance. J Cardiovasc Magn Reson.

[7] Schaafs L-A, Wyschkon S, Elgeti M (2020). Diagnosis of left ventricular diastolic dysfunction using cardiac magnetic resonance imaging: comparison of volume-time curves derived from long- and short-axis cine steady-state free precession datasets. Rofo.

[8] Chuang ML, Gona P, Hautvast GLTF (2012). Correlation of trabeculae and papillary muscles with clinical and cardiac characteristics and impact on CMR measures of LV anatomy and function. JACC Cardiovasc Imaging.

[9] Park E-A, Lee W, Kim H-K, Chung JW (2015). Effect of papillary muscles and trabeculae on left ventricular measurement using cardiovascular magnetic resonance imaging in patients with hypertrophic cardiomyopathy. Korean J Radiol.

[10] Patel AR, Mor-Avi V (2012). Are Trabeculae and papillary muscles an integral part of cardiac anatomy. JACC Cardiovasc Imaging.

[11] Duarte R, Fernandez-Perez G, Bettencourt N (2012). Assessment of left ventricular diastolic function with cardiovascular MRI: what radiologists should know. Diagn Interv Radiol.

[12] Bakir M, Wei J, Nelson MD, Mehta PK (2016). Cardiac magnetic resonance imaging for myocardial perfusion and diastolic function—reference control values for women. Cardiovasc Diagn Ther.

[13] Kawaji K, Codella NCF, Prince MR (2009). Automated segmentation of routine clinical cardiac magnetic resonance imaging for assessment of left ventricular diastolic dysfunction. Circ Cardiovasc Imaging.

[14] Maceira A, Prasad S, Khan M, Pennell D (2006). Normalized left ventricular systolic and diastolic function by steady state free precession cardiovascular magnetic resonance. J Cardiovasc Magn Reson.

[15] Krishnamurthy R, Pednekar A, Cheong B, Muthupillai R (2010). High temporal resolution SSFP cine MRI for estimation of left ventricular diastolic parameters. J Magn Reson Imaging.

[16] Aquaro GD, Pizzino F, Terrizzi A (2019). Diastolic dysfunction evaluated by cardiac magnetic resonance: the value of the combined assessment of atrial and ventricular function. Eur Radiol.

[17] Ashrafpoor G, Bollache E, Redheuil A (2014). Age-specific changes in left ventricular diastolic function: a velocity-encoded magnetic resonance imaging study. Eur Radiol.

[18] Garcia MJ, Thomas JD, Klein AL (1998). New Doppler echocardiographic applications for the study of diastolic function. J Am Coll Cardiol.

[19] Flachskampf FA, Biering-Sørensen T, Solomon SD (2015). Cardiac imaging to evaluate left ventricular diastolic Function. JACC Cardiovasc Imaging.

[20] Kawel-Boehm N, Hetzel SJ, Ambale-Venkatesh B (2020). Reference ranges ("normal values") for cardiovascular magnetic resonance (CMR) in adults and children: 2020 update. J Cardiovasc Magn Reson.

[21] Schoennagel BP, Fischer R, Grosse R (2015). Peak filling rates assessed by CMR imaging indicate diastolic dysfunction from myocardial iron toxicity. JACC Cardiovasc Imaging.

